# The Impact of the Methacrylation Process on the Usefulness of Chitosan as a Biomaterial Component for 3D Printing

**DOI:** 10.3390/jfb15090251

**Published:** 2024-08-30

**Authors:** Marta Klak, Katarzyna Kosowska, Milena Czajka, Magdalena Dec, Sylwester Domański, Agnieszka Zakrzewska, Paulina Korycka, Kamila Jankowska, Agnieszka Romanik-Chruścielewska, Michał Wszoła

**Affiliations:** 1 Foundation of Research and Science Development, 01-793 Warsaw, Poland; milena.czajka@polbionica.com (M.C.); magdalena.dec@polbionica.com (M.D.); korycka.paulina93@gmail.com (P.K.); kamila.jankowska2106@gmail.com (K.J.); agaromanik@wp.pl (A.R.-C.); michal.wszola@polbionica.com (M.W.); 2 Polbionica Ltd., 01-793 Warsaw, Poland; sylwester.domanski@polbionica.com (S.D.); agnieszka.zakrzewska@polbionica.com (A.Z.)

**Keywords:** biomaterial, chitosan, methacrylation, extracellular matrix, bioprinting, tissue engineering, tissue regeneration

## Abstract

Chitosan is a very promising material for tissue model printing. It is also known that the introduction of chemical modifications to the structure of the material in the form of methacrylate groups makes it very attractive for application in the bioprinting of tissue models. The aim of this work is to study the characteristics of biomaterials containing chitosan (BCH) and its methacrylated equivalent (BCM) in order to identify differences in their usefulness in 3D bioprinting technology. It has been shown that the BCM material containing methacrylic chitosan is three times more viscous than its non-methacrylated BCH counterpart. Additionally, the BCM material is characterized by stability in a larger range of stresses, as well as better printability, resolution, and fiber stability. The BCM material has higher mechanical parameters, both mechanical strength and Young’s modulus, than the BCH material. Both materials are ideal for bioprinting, but BCM has unique rheological properties and significant mechanical resistance. In addition, biological tests have shown that the addition of chitosan to biomaterials increases cell proliferation, particularly in 3D-printed models. Moreover, modification in the form of methacrylation encourages reduced toxicity of the biomaterial in 3D constructs. Our investigation demonstrates the suitability of a chitosan-enhanced biomaterial, specifically methacrylate-treated, for application in tissue engineering, and particularly for tissues requiring resistance to high stress, i.e., vascular or cartilage models.

## 1. Introduction

Recently, there have been significant advances in 3D bioprinting technology, particularly concerning the printing of tissues and organs. Increasingly, precise and comprehensive investigations are being undertaken to develop optimal biomaterials for suitable applications [1,2,3,4,5]. Since the origin of 3D bioprinting in the 1990s, scientists have made significant steps in the development and testing of various biomaterials specifically adjusted for 3D bioprinting applications. These biomaterials have undergone rigorous testing, demonstrating compatibility with the sophisticated demands of 3D bioprinting technology and exhibiting notable success in tissue engineering [2,6,7]. These biomaterials play a key role in the creation of functional and structurally sound tissues. They are chosen based on their ability to seamlessly integrate with the 3D printing process, maintain cellular structures, and induce the necessary biological responses within the organism [7]. Some notable examples of biomaterials used in 3D bioprinting include natural substances like gelatin [8], sodium alginate [9] derived from seaweed, and synthetic polymers such as polyglycolic acid (PGA) [10] or polylactic acid (PLA) [11]. Composites, combining different biomaterials, are also explored for enhanced structural and biomechanical integrity. The continuous improvement and expansion of biomaterial options contribute to the versatility and potential applications of 3D bioprinting in fields such as regenerative medicine, tissue replacement, and drug testing. Despite the relative youth of the technology, the robust testing and successful implementation of these biomaterials underscores the promising future of 3D bioprinting in advancing medical and scientific frontiers [2,3,6,7].

A substantial challenge is achieving the most suitable biomaterials adjusted to specific applications. Therefore, there is a growing interest in biomaterials susceptible to modification, either through chemical modification or the introduction of various types of additives to biomaterials [1,12,13,14]. This pursuit aims to enhance the functionality of these biomaterials and adjust them more precisely based on their designated objectives [1,14]. In addition to requirements pertaining to the fabrication of such biomaterials, parameters such as biocompatibility and biodegradability are of paramount importance [15,16]. Furthermore, in the quest for a well-defined biomaterial, fundamental characteristics must be considered, encompassing precise adaptation to the mechanical properties of tissue, appropriate rheological properties, good printability, favorable cell adhesion properties, and the extracellular matrix (ECM) of the tissue [17,18]. One of the noteworthy biomaterials that may serve as an additive to biomaterials, meeting the aforementioned criteria, is chitosan [1,19,20].

**Figure 1:**
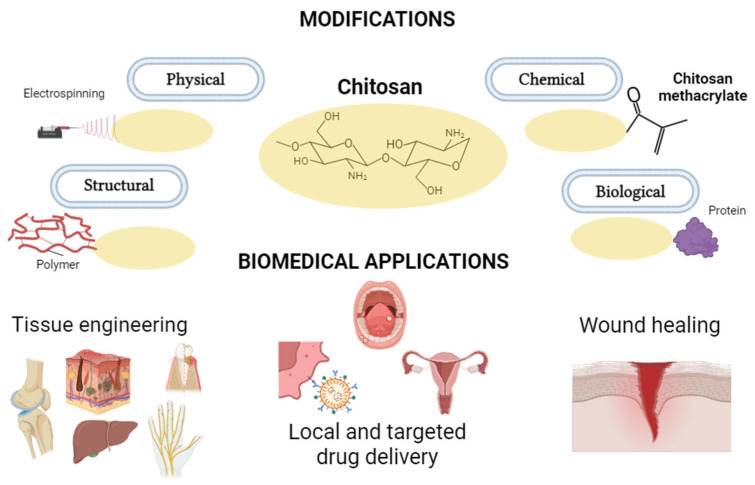
Diagram showing modifications of chitosan and its applications in medicine.

To date, chitosan has found application in the creation of scaffolds for the regeneration of various tissues, such as bone [21], cartilage [19], skin [19,22], nerves [22], periodontium [23], liver [24], and eardrum tissues [25] (Figure 1). Due to its versatility in applications, chitosan is considered a biomaterial with significant potential [1,25]. Chitosan is a naturally occurring polysaccharide derived from chitin, which is found in the exoskeletons of crustaceans such as shrimp, crabs, and other shellfish. It is created through the deacetylation of chitin, a process that removes the acetyl groups from the chitin molecule. Chemically, chitosan is a linear polysaccharide composed of repeating units of glucosamine and N-acetylglucosamine [1,26]. Chitosan, due to its structural similarity to glycosaminoglycans present in connective tissues, is an ideal material for applications in tissue engineering, especially in the bioprinting of blood vessels [1,19,27]. Chitosan is biocompatible, which means that it is well tolerated by living tissues, thus reducing the risk of adverse reactions. Furthermore, its biodegradability allows gradual degradation in the body, adapting to regenerative medicine principles requiring temporary support structures [1,19,22]. Chitosan can be used as an excellent scaffold for tissue regeneration that provides support for cell attachment, proliferation, and differentiation [27]. This is particularly crucial in regenerating damaged tissues such as bone, cartilage, and skin [19,21]. In addition, chitosan has been found to stimulate various healing processes. It can modulate the immune response and inflammatory reactions, promoting a conducive environment for tissue repair and regeneration [1,19,28]. Chitosan has inherent antimicrobial properties, which is particularly beneficial in medical applications where maintaining sterility is critical [1,19,22]. In 3D bioprinting, chitosan is used as a biomaterial to create complex three-dimensional structures. Its ability to form stable hydrogels makes it suitable for encapsulating cells and printing complex tissue-like structures [1,19,29]. Moreover, it has mucoadhesive properties making it suitable for drug delivery applications [30].

Researchers are attempting to modify existing biomaterials to increase their functionality. Chitosan is an ideal biomaterial for these purposes due to the presence of various chemical groups. Modifications of chitosan to improve its properties for 3D bioprinting can be divided into physical, structural, biological, and chemical [31] (Figure 1). Physical modifications include nanostructuring [32], i.e., its transformation at the nano level, improving its mechanical, surface, and biological properties, and cross-linking modifications. These affect the spatial structure of chitosan, regulating its durability and resistance [33]. Structural modifications, e.g., changes in the crystal structure of chitosan can affect its mechanical and degradation properties [34]. On the other hand, the modification of chitosan through the addition of other biomaterials allows for the creation of composites with enhanced properties. This strategy is widely employed to combine the favorable characteristics of chitosan with those of other materials, resulting in a synergistic effect. For this purpose, for example, the following materials can be used: nanoparticles (graphene) [35], natural polymers (collagen) [36], bioceramic additions such as silica [37] or biotech molecules, i.e., growth factors [38], peptides [39], and drugs [40,41]. The results of this modification enhance its capacity to stimulate various biological processes [35,36,37,38,39,40,41].

Among chemical modifications, the most important functional groups capable of modification in chitosan are the amino groups (-NH_2_). The presence of these reactive groups in chitosan guarantees its ability to form complexes with metal ions and retain water or interact with other molecules present in living organisms. Modifications of these amino groups frequently result in alterations to the surface charge of chitosan molecules, thereby influencing their properties and reactions with other substances [1,31]. Additionally, other chemical modifications include, e.g., hydroxyl group impact solubility, adhesion, and the ability to form bonds with other substances [1,42]. The esterification of chitosan, which involves the introduction of ethyl, methyl, or other alkoxy groups, has notable effects on the solubility and mechanical properties of chitosan-incorporated biomaterials [43]. Furthermore, chitosan is receptive to carboxylation, a chemical modification to introduce bioactive functions responsible for the controlled release of biologically active substances [44].

Methacrylation is a particularly beneficial modification of chitosan. Through the methacrylation process of the biomaterial targeted for bioprinting, the chemical, mechanical, and biological properties can be strategically influenced. A critical aspect of the bioprinting process is the ability of the chitosan methacrylate to improve the biomaterial’s ability to adhere to substrates or cells [1,19,45]. Additionally, it can exert control over mechanical properties, such as elasticity and strength, allowing customization to specific application requirements, particularly in the biomechanics of printed tissues [1,46]. Furthermore, the introduction of methacrylate groups can influence the solubility of the biomaterial, which is a critical consideration during the bioprinting process, especially when various solvents are employed. The incorporation of methacrylated groups presents possibilities for further chemical modifications, enabling precise tailoring of the biomaterial for specific applications, notably concerning the controlled release of bioactive substances. Moreover, the advantages of methacrylation, particularly in biomedical applications, extend to potential effects on biocompatibility with the organism. It can also impact the surface characteristics of the biomaterial, influencing interactions with cells, proteins, and other biological components [1,45,46,47].

The purpose of this study was to assess the impact of methacrylation on chitosan physicochemical parameters. The novel element of this research is the proprietary composition of the biomaterial under study containing dECM, methacrylated gelatin, methacrylated hyaluronic acid, and chitosan or methacrylated chitosan. The composition of the material under study is covered by a patent. The essence of this work was to identify the functional differences of the tested materials differing in the content of chitosan or methacrylated chitosan. The experimental investigation included the evaluation of functional properties, such as rheology, printability, mechanical strength, elasticity, degradation, soaking, and swelling. In addition, biocompatibility studies were performed to evaluate their biological properties from the perspective of using biomaterials in tissue engineering.

## 2. Materials and Methods

### 2.1. Materials

#### 2.1.1. Biomaterials Components

The biomaterials were prepared as a composition of three main ingredients: (i) decellularized pancreatic extracellular matrix hydrogel (Printiss^®^ dECM-PAN; Polbionica Ltd., Warsaw, Poland), the concentration of dECM in the final biomaterial composition is 76.6 mg/mL; (ii) hydrogel methacrylated gelatin (TINTBIONIC GELMA 80; Polbionica Ltd., Warsaw, Poland) in PBS with a concentration in the final biomaterial composition of 31 mg/mL and methacrylated hyaluronic acid (TINTBIONIC HAMA; Polbionica Ltd., Warsaw, Poland) with PBS with a final concentration in the biomaterial of 3.1 mg/mL with LAP (lithium phenyl-2,4,6-trimethylbenzoylphosphinate) (Polbionica Ltd., Warsaw, Poland) as a photoinitiator with a final concentration of 1.85 mg/mL; (iii) methacrylated chitosan (TINTBIONIC CHIMA; Polbionica Ltd., Warsaw, Poland) dissolved in 1% acetic acid (Sigma-Aldrich, Taufkirchen, Germany) and neutralized with a final concentration in the biomaterial of 3.1 mg/mL or chitosan (Sigma-Aldrich, Taufkirchen, Germany) dissolved in 2% acetic acid (Sigma-Aldrich, Taufkirchen, Germany) and neutralized with a final concentration in the biomaterial of 1.4 mg/mL. The improved biomaterials were designated as chitosan biomaterial (BCH) and biomaterial containing methacrylated chitosan (BCM). Both biomaterials were additionally enriched with glycerol at a concentration of 89.0 mg/mL.

#### 2.1.2. Cell Culture

The mouse fibroblast cell line L-929 (ATCC^®^, CCL-1TM, Manassas, VA, USA) was used. The L-929 cells were cultured in Dulbecco’s Modified Eagle Medium (ATCC^®^, Manassas, VA, USA) with 10% fetal bovine serum (Sigma-Aldrich^®^, St. Louis, MI, USA) and penicillin–streptomycin solution (Corning ™, Gilbert, AZ, USA).

The RFP Expressing Human Dermal Fibroblasts-Neonatal cell line (RFP-HDFCs-Neo, ANGIO-PROTEOMIE, cAP-0008-NeoRFP, Boston, MA, USA) was used. The RFP-HDFCs-Neo cells were cultured in Dulbecco’s Modified Eagle Medium (ATCC^®^, Manassas, VA, USA) supplemented with 15% fetal bovine serum (Sigma-Aldrich^®^, St. Louis, MI, USA) and penicillin–streptomycin solution (Corning ™, Gilbert, AZ, USA).

All cell lines were incubated at 37 °C in a humidified 5% CO_2_ atmosphere to ensure optimal growth and maintenance.

### 2.2. Methods

#### 2.2.1. Preparation of CHIMA

**Figure 2:**
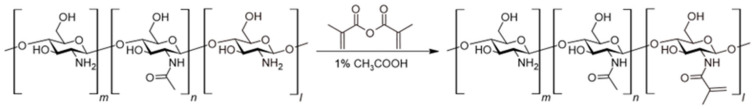
The scheme of the methacrylation process.

A 1000 mL three-necked flask equipped with a stirring bar was placed in a heating block on a magnetic stirrer. An amount of 500 mL of 1% CH3COOH (acetic acid solution in water) was measured using a measuring cylinder and poured into the flask. One side neck was secured with a septum, and a thermocouple was installed in the other. A reflux condenser was placed in the center and the water flow was started. The flask was protected from light and heating was started to the set temperature of 50 °C. An amount of 5.0 g of chitosan was weighed on an analytical balance and added in portions to the flask, mixing its contents at a speed of 1000–1200 rpm. After adding the all-weighted material, it was stirred overnight to completely dissolve the powder. A 1% (*w*/*v*) solution with pH1 = 4.02 was obtained. Then, 17.5 mL of methacrylic acid anhydride (MAA) was taken into a 20 mL syringe and instilled into the solution using a syringe pump at a set rate of 5.9 mL/h. The total instillation time was about 3 h. The reaction mixture was left overnight, with continuous stirring and heating of the reaction system (pH2 = 3.06). Next, the reaction mixture was diluted with 2000 mL of PBSx1 to obtain a 5-fold dilution and neutralized with 1000 mL 1M NaHCO_3_ to obtain pH ≈ 7.00. The solution was transferred into dialysis tubes (membrane MWCO = 12–14 kDa) and placed in 5 L beakers filled with demineralized water. The beakers were protected with aluminum foil from excessive exposure to light and placed on magnetic stirrers, heating to 40 °C and stirring at 400 rpm. The dialysis process was carried out at this temperature for 5 days, changing the water twice a day (10 water changes in total). After the dialysis process was completed, the solution was concentrated to a final volume of about 1000 mL on a rotary evaporator at 45 °C, under a pressure of about 40–70 mbar. The solution was frozen at −80 °C and lyophilized at 10 °C under a pressure of 0.10 mbar for 48 h to obtain pure methacrylated chitosan (CHIMA) as shown in Figure 2.

#### 2.2.2. Degree of Substitution

A 2 mg sample of methacrylated chitosan (CHIMA) was dissolved in 600 μL of D2O with the addition of 0.00916 mmol of TMSP (quantitative and chemical shift standard) and placed in a 5 mm NMR tube. Deuterated acetic acid was then added to reach a concentration of 1%. The samples were then placed in an NMR spectrometer (Agilent DirectDrive2 700 MHz). The temperature was set to 60 °C. After the temperature stabilized, the samples were rotated, the probe was tuned, the pulse was measured, and the magnetic field inhomogeneity was corrected. Then, the 1H spectrum was measured (measurement parameters: number of scans 8, repetition time 15 s, and pulse time 45° 2.5 μs).

The spectrum was analyzed using the NMRGlue package in the Python environment. After importing the data, exponential weighting (line broadening: 2 Hz), Fourier transforms, phasing, and baseline correction were performed for the regions 5.85 ppm: 5.6 ppm, 3.3 ppm: 3.05 ppm, and 0.1 ppm: −0.1 ppm.

Then, the integrals of the peaks in the region of 5.85 ppm: 5.65 ppm (corresponding to protons from methacrylic groups) were calculated and the peak in the region of 3.3 ppm: 3.0 ppm (the peak of the proton coming from merium) was integrated. The method of spectrum analysis is analogous to that presented for methacrylated gelatin [48], with the modification related to the fact that CHIMA methacrylation occurs only in one position; we expect two (and not six) peaks in the region of methacrylic protons =CH_2_. We integrated one of them and compared it with the reference peak of one of the mer protons. Based on these parameters, the DSNMR value (degree of substitution) was calculated using the following formula:

(1) $${DS}_{NMR}=\frac{\int peak\;(5.85-5.65\;ppm)}{\int peak\;(3.3-3.0\;ppm)}\cdot 100\%$$

#### 2.2.3. Rheology

The rheological properties of dECM biomaterials with the addition of CH or ChiMa were assessed using MCR 72 (Anton Paar, Warsaw, Poland). For this purpose, the dynamic viscosity was measured at 20 °C temperature at a shear rate of 100/s. The complex modulus was also measured for a temperature of 20 °C at a constant frequency of 1 Hz and a variable amplitude of 0.1–100%.

#### 2.2.4. Printability

**Figure 3:**
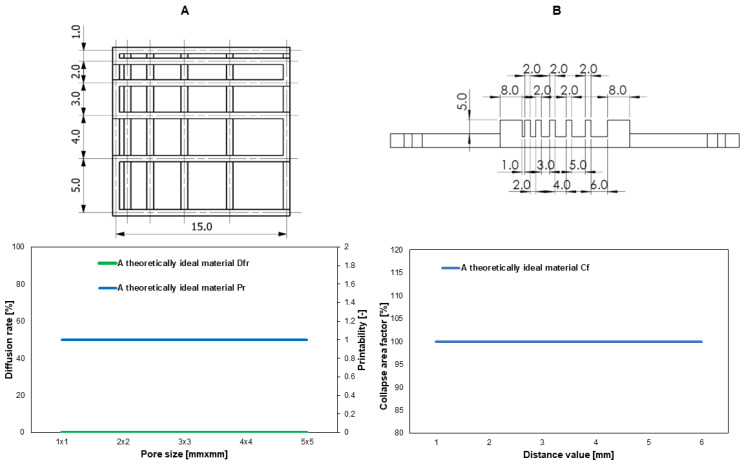
The models of printable structures to evaluate printability in (**A**) the fiber fusion test and (**B**) the fiber collapse test.

The printability of the materials was evaluated with the following tests: a fusion test of fibers printed in the form of a template, a collapse test of a fiber printed on a 3D platform, and a continuity test of fibers printed from the tested biomaterial in a volume of 3 mL. The method was taken from the available literature [49,50,51]. Figure 3 shows the template and platform model. Additionally, the dependencies of the assessed parameters for a theoretically ideal material are shown.

The fiber fusion test involves printing using the test material according to the designed model of two layers printed one after the other without using cross-linking between them. The prints were made using a BIO X™ printer (Cellink, Gothenburg, Sweden). The 2D print prepared has a 0–90° pattern, and the distance between the fibers ranged from 1 to 5 mm and increased in 1 mm step increments. The template model is presented in Figure 3. It was printed using the following conditions: printing speed of 20 mm/s, needle diameter of 0.609 mm or 0.437, and printing distance of 0.8 mm. During the test, the test material was extruded at the appropriate range of pressures and temperatures. The print was cross-linked using an external UV-Vis 405 nm lamp with a POLBIONICA UV VIS lamp (Polbionica, Warsaw, Poland) for 15 s at 13 W/cm^2^. Images were processed using Carl Zeiss Vision AxioVision Viewer 4.8 software (Carl Zeiss Vision GmbH, Warsaw, Poland). From the results, two parameters described by equations were determined, i.e., percentage diffusion (flow) rate (*Df_r_*) and printability (*P_r_*). The degree of pore diffusion without material spreading is 0 (i.e., *A_t_* = *A_a_*), and for a perfect model reproduction, the printability is 1.

(2) $${Df}_{r}=\frac{A_{t}-A_{a}}{A_{t}}\cdot 100\%$$

(3) $$P_{r}=\frac{L_{a}^{2}}{16\cdot A_{a}}$$

The above equations were used to determine the pore diffusion rate *Df_r_* and the printability *P_r_* of the material, where

*A_t_*—theoretical pore surface area;
*A_a_*—the actual surface area of the pore;
*L_a_*—the actual perimeter of the pore.

To determine the collapse of the material, the mid-span deflection of the suspended fiber was analyzed. For the experiment, a special platform consisting of seven pillars offset from each other by known distances of 1, 2, 3, 4, 5, and 6 mm was designed and 3D-printed. The dimensions of the five pillars placed inside the structure are 2 × 10 × 6 mm^3^, and the dimensions of the two edge pillars are 5 × 10 × 6 mm^3^. The platform model is presented in Figure 3.

The fiber of the material under test was deposited on the platform, and the print image was taken immediately. The temperature and pressure conditions during the test were adjusted to the test material, and the print was made at a speed of 20 mm/s with a 0.609 mm needle. The refractive area index (*C_f_*) was calculated using the following equation:

(4) $$C_{f}=\frac{A_{a}^{c}}{A_{t}^{c}}\cdot 100\%$$

The above equation was used to determine the collapse area factor (*C_f_*), where

*A_a_^c^*—the actual area under the curve;
*A_t_^c^*—the theoretical area under the curve.

Fiber continuity when printing with 2–3 mL of the tested biomaterial, 0/1 system: interrupts the fiber—0; continues—1.

#### 2.2.5. Mechanical Testing

The mechanical compressive strength of the samples was tested using a static compression test. For the analysis, cylindrical specimens with dimensions of d, diameter of 10 mm, and h, height of 5 mm, were designed and printed using a BIO X™ printer (Cellink, Gothenburg, Sweden) with 100% filling and cross-linking using an external UV-Vis lamp after each layer. The samples were statically compressed at a constant speed of 10 mm/min at room temperature until 80% strain was reached, and points were collected every 0.025 s. The value of the calculated mechanical strength of the specimens was analyzed as the maximum shear stress (the ratio of the compressive force to the surface area of the printed specimen) and Young’s modulus as the slope coefficient of the simple stress–strain relationship of the specimen in the deformation range of 0.1–0.5 [-]. Another parameter evaluated was the conventional elastic limit, i.e., the stress required to deform the sample by 10%.

#### 2.2.6. Degradation

Biomaterial samples in a volume of 300 µL were cross-linked with UV light (365 nm, 15 s, 13 mW/cm^2^) and immersed in SBF (simulated body fluid) solution with and without collagenase (0.1 mg/mL; Sigma-Aldrich, Germany). After 21 days, the samples were lyophilized and weighed (*W_d_*). The weight of the biomaterial at 0 h was a cross-linked sample after lyophilization (*W*_0_). The experiment was performed in 3 repetitions. The degree of degradation (*O_d_*) was calculated from the following equation:

(5) $$O_{d}=\frac{W_{0}-W_{d}}{W_{0}}\cdot 100\%$$

#### 2.2.7. Effective Swelling and Absorbability

The swelling experiment was carried out on constructs made of hydrogels and biomaterials containing different ratios of GelMa, HaMa, and Chitosan or ChiMa. After photopolymerization by UV radiation (13 mW/cm^2^, 15 s, 365 nm), the samples were lyophilized, weighted (*W*_0_), and then immersed in deionized water at room temperature. The swelling weight (*W_s_*) was measured after 24 h. The degree of swelling *O_t_* was calculated according to the following equation:

(6) $$O_{t}=\frac{W_{s}-W_{0}}{W_{0}}\cdot 100\%$$

The water absorption test was carried out analogously to the swelling test. The cross-linked biomaterial samples were weighed (*W*_0_) and directly flooded with deionized water. At 3 time points (24, 48, and 72 h), unabsorbed water was collected and the samples were reweighed (*W_a_*). The degree of water absorption (*O_a_*) was calculated from the following equation:

(7) $$O_{a}=\frac{W_{a}-W_{0}}{W_{0}}\cdot 100\%$$

The experiment was performed in 3 replications for each biomaterial.

#### 2.2.8. The Assessment of Cell Biocompatibility Following Model Bioprinting and Cell Culture on the Surface of Biomaterials

##### Preparation and Bioprinting of Cell-Laden Biomaterial

In this study, two biomaterial variants, BCH and BCM, were prepared. Cell biocompatibility was evaluated and both post-bioprinting and RFP-HDFCs-Neo were employed for the investigation. The cell-laden biomaterial formulations included RFP-HDFCs-Neo at a density of 2 × 10^6^ cells/mL. The cell-laden biomaterials were loaded into sterile CELLINK syringes for subsequent bioprinting. The G-code was imported into the Bio X 3D bioprinter, and biomaterial was dispensed through a 21 G needle, employing extrusion-based bioprinting at a speed of 20 mm/s, a temperature of 22 °C, and a pressure of 25 kPa. The bioprinted construct was cultured at 37 °C and 5% CO_2_ in a culture medium dedicated to the cell line. The biocompatibility of cells in the bioprinting model was evaluated 24 h post-bioprinting. In addition, a positive control was performed, which consisted of cells seeded onto culture dishes and cultured under standard conditions, i.e., 37 °C and 5% CO_2_.

##### Preparation and Cell Seeding on the Surface of Biomaterials

In this study, two biomaterial variants, BCH and BCM, were used. Cell biocompatibility was assessed as the result of the interaction between cells and the biomaterial surface 24 h post-seeding. The cells were observed and analyzed for coverage on each biomaterial. Prior to seeding the cells onto a biomaterial, the 24-well plate was coated with an approximately 1 mm thick layer of each biomaterial variant given and then cross-linked. In the experiment, RFP-HDFCs-Neo were used at a density of 3 × 10^4^ cells/well. The cells seeded on the surface were cultured at 37 °C and 5% CO_2_ in a culture medium dedicated to the cell line. The positive control was cells cultured in culture dishes under 37 °C and 5% CO_2._

##### Microscopic Image Analysis

Cell imaging in both printed models and cell seeding on the surface of biomaterials was conducted using the inverted fluorescent microscope Olympus IX83 (Olympus, Tokio, Japan). The imaging process involved the utilization of 2×, 4×, and 10× objectives in both bright field (BF) and fluorescent light modes, specifically employing tetramethylrhodamine (TRITC) filters. The images were performed 24 h after printing or seeding. The acquired images were subjected to editing using Olympus cellSens2010 software for enhanced visualization and analysis. This comprehensive imaging approach allowed for detailed observation and documentation of cellular morphology, distribution, and interactions within the printed models and on the biomaterial surfaces.

##### Cytotoxicity Assay

The LDH-Glo™ Cytotoxicity Assay (Promega, Madison, WI, USA) was employed to assess the direct cytotoxicity of the biomaterials (BCH and BCM) in the RFP-HDFCs-Neo cells. This evaluation was conducted in printed models and conventional cell cultures on the surface of the respective biomaterials. The assay was conducted 24 h after printing and seeding. In the experiment, the culture medium was collected and diluted at a ratio of 1:100 in LDH Storage Buffer following the manufacturer’s instructions. Subsequently, all collected samples were stored at −20 °C until the time of the test. The LDH assay was conducted in accordance with the manufacturer’s protocol. After a one-hour incubation period at room temperature, the luminescence signal was measured using a microplate reader (Agilent BioTek Synergy H1 Plate Reader, Agilent Technologies, Inc. Headquarters, Santa Clara, CA, USA). This test offers valuable insights into the cytotoxicity arising from interactions between cells and biomaterials over a specific period. In addition, a control of the reaction was performed. The negative control was cells cultured under standard conditions, i.e., 37 °C and 5% CO_2_ and biomaterials without cells. The positive control was cells and biomaterials with cells cultured under standard conditions exposed to one hour of incubation with 0,1% Triton X-100.

An evaluation of the effects of biomaterial extracts on L-929 (in accordance with standard PN-EN ISO 10993-5:2009) [52] and RFP-HDFCs-Neo cells was also performed using an independent experiment test. The biomaterials (BCH and BCM) were fragmented using a sterile scalpel into 10–15 smaller pieces. Extracts from fragmented biomaterials were prepared by inserting them into Eppendorf-type tubes. A dedicated culture medium was poured at a ratio of 0.2 g of biomaterial and 1 mL of medium. This was subsequently incubated for 24 h at 37 °C with 5% CO_2_. Then, extracts, along with a positive control (CP, 0.1% Triton/DMEM), were added to cells seeded in 96-well plates at a density of 1 × 10^3^ cells per well. The cells were incubated at 37 °C with 5% CO_2_ in a cell medium for 24 h. After the incubation period, the medium was collected and the test was performed according to the manufacturer’s protocol.

##### Cell Proliferation

Cell proliferation of RFP-HDFCs-Neo, both in printed models and those directly cultured on the surfaces of biomaterials (BCH and BCM), was assessed using the Alamar Blue assay (Invitrogen™, Waltham, MA, USA). The experimental protocol entailed a 24 h incubation of cells with the Alamar Blue reagent at a 1:10 ratio under standard culture conditions, i.e., 37 °C and 5% CO_2_. Subsequent to this incubation period, 100 µL of the medium was transferred to black plates to absorb light, minimizing background interference and crosstalk. The absorbance of each sample was then measured at 530 nm and 590 nm using a microplate reader (Agilent BioTek Synergy H1 Plate Reader, Agilent Technologies, Inc. Headquarters, Santa Clara, CA, USA). This methodology yields crucial insights into cell proliferation dynamics in printed models and on biomaterial surfaces at distinct time points, elucidated through changes in absorbance values at specific wavelengths. The negative and the positive control of the reaction was performed. The controls in both were the same as in the assay above.

In addition, an assessment of the effect of biomaterial extracts on cells of L-929 (in accordance with standard PN-EN ISO 10993-5:2009) [52] and RFP-HDFCs-Neo was performed. Extracts were prepared as above and the Alamar Blue assay was carried out according to the manufacturer’s protocol.

##### Gene Expression

The expression levels of specific genes were investigated using Real-Time PCR gene expression analysis. Total RNA was isolated using TRI Reagent Solution™ (Invitrogen™, Waltham, MA, USA). The purity of the RNA was assessed using the NanoDrop™ One/OneC Microvolume UV-Vis Spectrophotometer (ThermoFisher SCIENTIFIC™, Waltham, MA, USA) by measuring absorbance at 260 nm and 280 nm wavelengths. All samples were removed from DNA using the DNA-free™ DNA Removal Kit (Invitrogen™, Waltham, MA, USA) following the manufacturer’s instructions. Reverse transcription was carried out using the High Capacity RNA-to-cDNA kit (Applied Biosystems™, Waltham, MA, USA) according to the manufacturer’s instructions. Gene expression analysis was performed using TaqMan^®^ gene expression assays (ThermoFisher SCIENTIFIC™, Waltham, MA, USA) for genes including *CD146* (Hs00174838_m1), *CD31* (Hs01065279_m1), and *VEGF-A* (Hs00900055_m1). All samples were analyzed in duplicates using 100 ng of total RNA per sample. Real-Time PCR was performed using a CFX96 Touch Real-Time PCR Detection System instrument (Bio-Rad, Hercules, CA, USA). The results were normalized to the GAPDH housekeeping gene. The relative gene expression was calculated using the 2^−ΔΔCt^ method [53].

#### 2.2.9. Statistical Analysis

The significant difference from the respective controls for each experimental test condition was assessed with a one-way analysis of variance (ANOVA) and the Dunnett test. The difference was significant if the *p*-value was less than 0.05. Statistical analysis was performed using GraphPad Prism V5.01 software (GraphPad Software Inc., La Jolla, CA, USA).

## 3. Results

### 3.1. Degree of Substitution and ^1^H NMR Analysis

**Figure 4:**
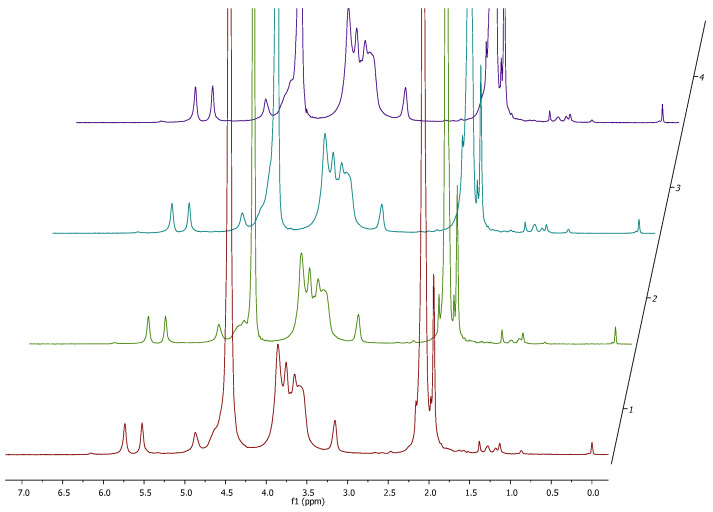
Stack plot of ^1^H NMR spectra collected for representative samples of CHIMA.

Methacrylated chitosan was obtained in a classical approach [54] employing methacrylic anhydride (MAA) as an acylating agent in a reaction carried out in 1% acetic acid. The use of an acidic environment is necessary to obtain satisfactory solubility of chitosan, which, however, has a negative impact on the reactivity of free amino groups due to their protonation. The protonated amino group of -NH_2_ loses its nucleophilic properties and transforms into -NH_3_^+^. Both forms remain in constant equilibrium in the reaction environment, which allows for its functionalization using a large excess of MAA. Through optimization work taking into account the amount of MAA used, temperature, and reaction duration, the target product with a degree of substitution DS ~ 80% was obtained. ^1^H NMR spectra were measured for each of the obtained products and the results are summarized in Figure 4. On their basis, the degree of product substitution was determined, which was 76% ± 5% for Sample 1, 82% ± 5% for Sample 2, 78% ± 5% for Sample 3, and 79% ± 5% for Sample 4. Additionally, it was demonstrated that there was no contamination with methacrylic acid residues, the signals of which are not visible in the obtained spectra. This result confirms the effectiveness of the method used to purify the reaction mixture. The finished and characterized product, according to the described procedure, was used for further research.

### 3.2. Rheology

**Figure 5:**
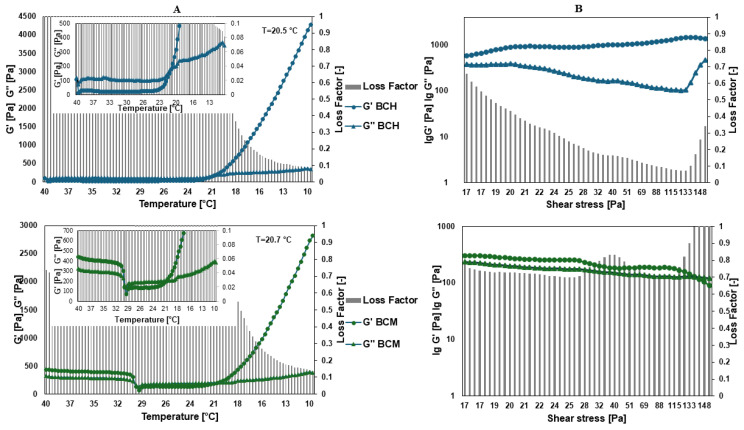
Results of rheology testing, where (**A**) is the dependence of the complex modulus on temperature and (**B**) is the complex modulus for BCH (biomaterial with chitosan) and BCM (biomaterial enriched with methacrylated chitosan).

Figure 5 shows the rheological characteristics of the obtained variants. The values of the storage modulus G′ for BCH in the tested amplitude range are in the range of 570.23–1462.4 Pa, and the loss modulus G″ is 101.97–461.11 Pa. The values of the storage modulus G′ for BCM in the tested amplitude range are in the range of 90.71–306.77 Pa, and the loss modulus G″ is 121.01–238.12 Pa. Based on the examination of the storage modulus, it can be observed that for all the samples tested, the storage modulus was larger than the loss modulus, which indicates that elastic properties predominate over viscous ones. For the BCH variant, a wide range of linear elastic viscosity can be observed, which means that the tested materials are stable in the tested range of oscillation amplitude. Variants concerning methacrylated chitosan BCM have a much lower value of storage modulus compared to BCH, and for a shear strain value of approximately 0.7 the characteristics change and the loss modulus is higher than the storage modulus, which means that viscous properties begin to be more important than elastic properties.

The viscosity of BCH is 122.07 (±45.18) mPa·s and for BCM it is 398.42 (±17.65) mPa·s. Variants with the addition of BCH have a viscosity value approximately three times lower than those with the addition of methacrylated chitosan. The study of the dependence of the complex modulus on temperature showed that the gelation temperature of all variants is in the range of 20.5–20.7 °C.

### 3.3. Printability

**Figure 6:**
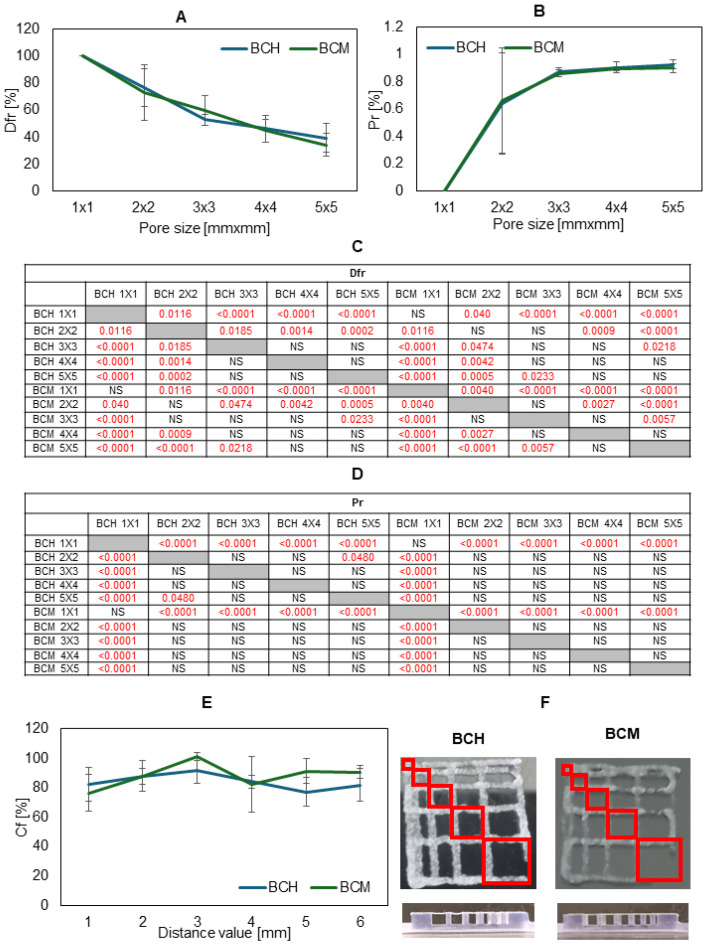
Results of printability testing, where (**A**) is the diffusion rate, (**B**) is the printability, and (**E**) is the collapse area factor for BCH (biomaterial with chitosan) and BCM (biomaterial enriched with methacrylated chitosan). In picture (**F**), the photos of printed models are shown. Tables (**C**,**D**) present the statistical analysis of individual variants of printability experimental results. Statistically significant values are marked in red. In the case of the collapse area factor, the differences were statistically significant for BCM 3 vs. BCH 1 (*p* = 0.029), BCM 3 vs. BCH 4 (*p* = 0.0461), BCM 3 vs. BCH 5 (*p* = 0.0060), BCM 3 vs. BCH 6 (*p* = 0.0023), BCM 3 vs. BCM 1 (*p* = 0.0046), and BCM 4 vs. BCM 3 (*p* = 0.0274). For other data sets, the differences were statistically insignificant.

Figure 6 shows the results of the usability test of the tested materials in 3D bioprinting technology.

The dependencies of factors such as the material diffusion rate and printability on the pore size on the printed template and the collapse coefficient of the fiber printed on the platform on the distance between the pillars were presented. The fiber collapse coefficient decreases as the pore size on the template increases, and thus the printability increases. The rate of spreading of the material printed in the form of a template model in the fiber fusion test for a pore size of 4 mm^2^ is lower for the BCM material than for the BCH material, which means that by using methacrylated chitosan as a biomaterial component, high mapping accuracy and high resolution can be achieved. The printability of both materials is satisfactory, the parameter for pores above 4 mm^2^ is over 0.8. The fiber collapse coefficient is more stable for BCM than BCH. For a distance of over 3 mm, a stable fiber stretched on the platform pillars was obtained. Similar conclusions can be drawn based on the results of the printable filament collapse test on the platform. The most stable fiber was obtained for the BCM material. All tested materials demonstrate fiber continuity and smoothness. For the BCH material, a smooth and continuous fiber was obtained at a temperature of 24 °C and an extrusion pressure of 60 kPa, while for the BCM material, the optimal printing parameters are a temperature of 25 °C and a pressure of 55 kPa.

### 3.4. Mechanical Testing

**Figure 7:**
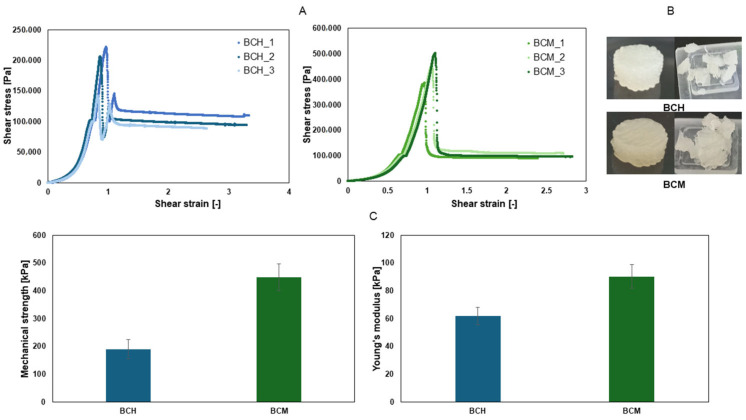
Results of mechanical testing. (**A**) The dependence of stress on the strain of test samples: blue color indicates BCH (biomaterial with chitosan) and green color indicates BCM (biomaterial enriched with methacrylated chitosan). (**B**) Photos of the printed and destroyed BCH and BCM structures. (**C**) mechanical parameters: the blue color indicates the mechanical strength values and the Young’s modulus values for BCH (biomaterial with chitosan) and the green color indicates the mechanical parameters for BCM (biomaterial enriched with methacrylated chitosan).

As a result of the work carried out, three cylindrical samples were printed from each tested material and subjected to a static compression test in order to obtain the mechanical parameters of the printed object. Based on the results obtained, the average mechanical strength and average Young’s modulus were determined for the tested samples, and the results are summarized in Figure 7.

Materials containing chitosan are characterized by lower values of mechanical parameters than materials containing methacrylated chitosan. The strength of the BCH material is two times lower than the strength of the BCM material. The material containing chitosan after methacrylation has significant elasticity, which can be used when printing constructs exposed to stress, i.e., tissue models with a vascular system or parts of cartilage.

### 3.5. Degradation, Absorbability, and Swelling Ratio

**Figure 8:**
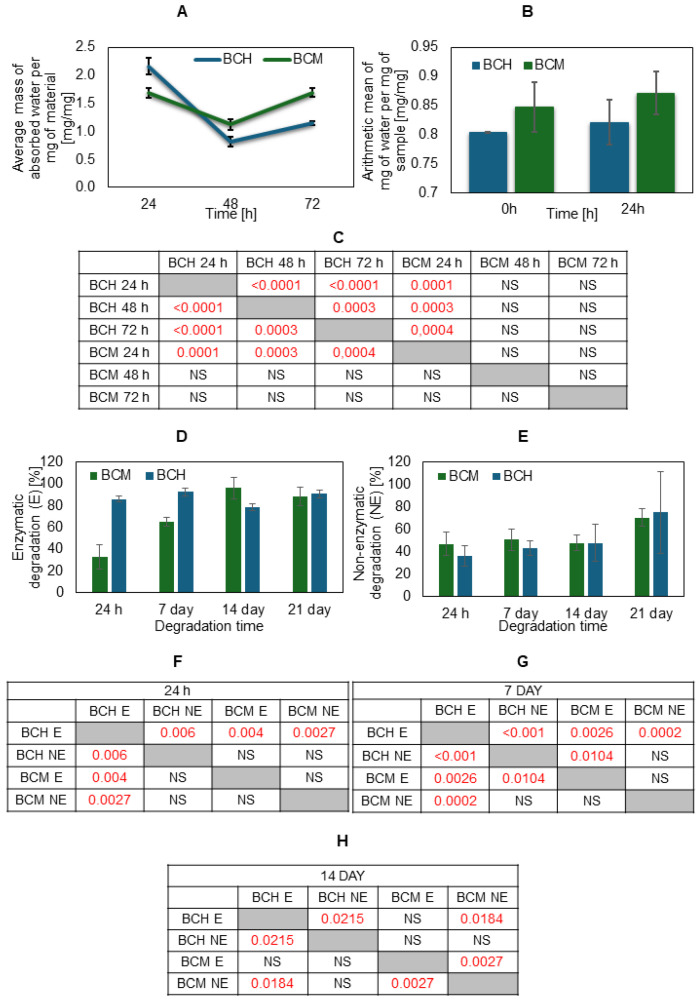
Results of water absorption, swelling test, and the degree of degradation for the tested materials. The average mass of mg of water per mg of sample in the (**A**) water absorption and (**B**) swelling test for BCH (biomaterial with chitosan) and BCM (biomaterial enriched with methacrylated chitosan); enzymatic degradation (**D**) after 24 h, 7 days, 14 days, and 21 days; and non-enzymatic degradation result (**E**) after the same. The tables (**C**,**F**–**H**) present the statistical analysis of individual variants of the water absorption experimental results and degradation after time. Statistically significant values are marked in red. In the case of the swelling test, the differences were statistically insignificant.

Analyzing water absorption at three time points, it can be seen that the highest water absorption per mg of sample is observed after 24 h, and in the following days (48 and 72 h) the amount of water absorbed decreases and the material becomes completely saturated with water (Figure 8). On the first day, the material containing methacrylated chitosan absorbed more water than the material containing chitosan, but on each subsequent day, the trend was reversed. In the case of the swelling test (Figure 6B), no statistical differences between the biomaterial variants are visible at a given time point. After freeze-drying, the material containing methacrylated chitosan absorbs more water than the material enriched with chitosan, both at time 0 and after 24 h.

It can also be seen that the use of methacrylated chitosan in biomaterials results in an increase in the degree of swelling compared to biomaterials with chitosan.

The degree of degradation for the tested materials is similar (Figure 8D,E). Enzymatic degradation (E) after 21 days causes almost complete degradation of the biomaterials, which amounted to approximately 90%—for BCH 91% and for BCM 82%. However, non-enzymatic degradation (NE) showed a degradation rate above 60%—for BCH 62% and for BCM 66%.

### 3.6. Assessment of Biocompatibility of Cells Cultured on Biomaterial Surfaces and 3D-Printed Models

#### 3.6.1. Microscopic Imaging Analysis

**Figure 9:**
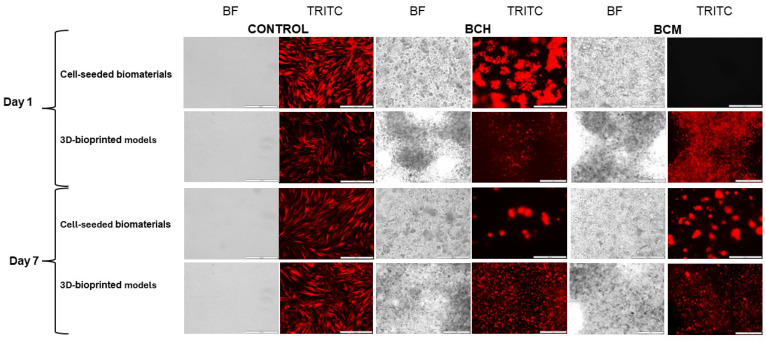
Microscopic imaging of RFP-HDFCs-Neo cells cultured on biomaterials and as a test model of printed constructs. Imaging was performed 24 h and 7 days after preparation of the constructs.. The images were made with an Olympus IX83 microscope (Olympus, PA, USA) in the bright field (BF) and with the use of red fluorescent lights (TRITC-tetramethylrhodamine). Imaging was performed at both 10× (cell-covered biomaterials) and 4× (3D-printed models) objective magnifications. Control: cells cultured under standard conditions (37 °C and 5% CO_2_); BCH: cells cultured on biomaterial with added chitosan; and BCM: cells cultured on biomaterial with added methacrylated chitosan.

In this study, a microscopic analysis of the RFP-HDFCs-Neo cells was performed. The organoleptic examination was conducted both in cell culture on biomaterials and in three-dimensional prints. The biomaterials in both examinations included cells and biomaterials with chitosan (BCH) and methacrylated chitosan (BCM). Microscopic imaging was performed 24 h and 7 days after the biomaterials were seeded with the cells or 3D-printed. The control of the experiment was cells maintained in adherent culture under standard conditions (37 °C and 5% CO_2_). Based on the microscopic evaluation performed, the presence of cells both cultured on biomaterials (cell-seeded biomaterials) and after the printing process (3D-bioprinted models) was observed. Cell-seeded biomaterial (BCH) cultured cells were noted under fluorescent light (TRITC). In 3D-bioprinted models, it was difficult to observe individual cells due to the translucency of the material. However, it was possible to observe cells forming aggregates. According to microscopic analysis for methacrylated biomaterial (BCM, cell-seeded biomaterials) 24 h after cell seeding, no cells were detected. Our preliminary studies show that increased cell proliferation on the BCM biomaterial occurs around seven days of culture. Three-dimensional-bioprinted models were observed, showing cells under TRITC fluorescence light in both bioprinted chitosan (BCH)- and methacrylated chitosan (BCM)-based biomaterials. In the printed models, a higher number of cells was observed in the models printed with the addition of methacrylated chitosan (BCM, 3D-bioprinted models), as seen in Figure 9, indicated by red beads.

#### 3.6.2. Cytotoxicity Assay

**Figure 10:**
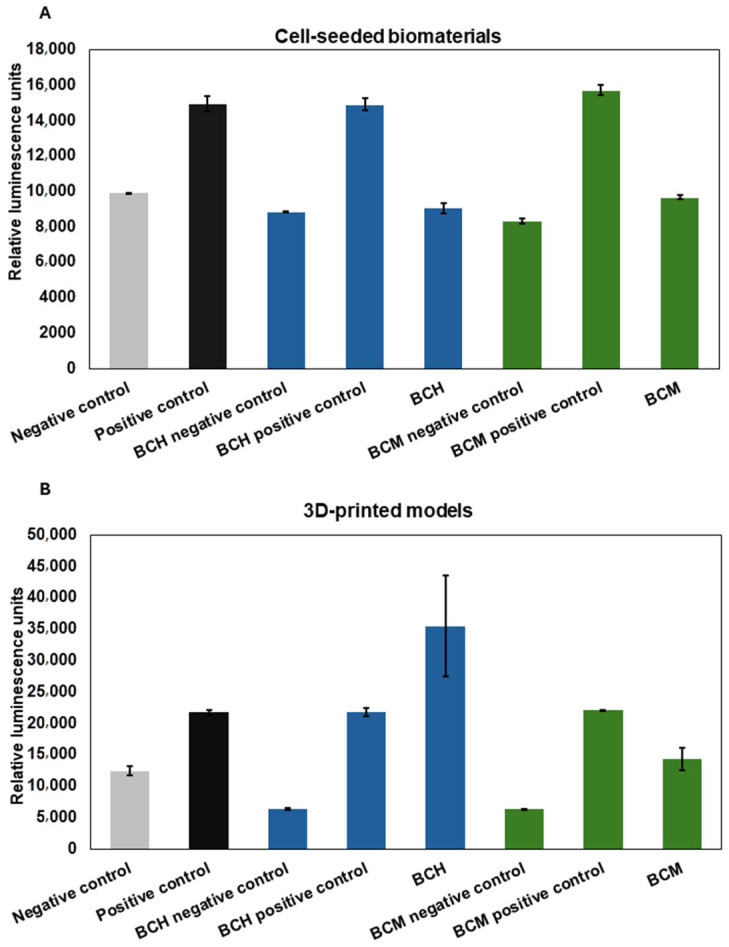
Cytotoxicity of biomaterials. LDH assay was performed as a result of the contact of cells with biomaterials ((**A**) cell-seeded biomaterials) and after the bioprinting process ((**B**) 3D-printed models). Negative control: cells cultured under standard conditions; positive control: cells cultured under standard conditions exposed to 0.1% Triton X-100; BCH: cells+ chitosan-biomaterial; BCM: cells+ methacrylated chitosan-biomaterial; BCH/BCM negative control: biomaterials without cells; and BCM/BCH positive control: biomaterials+ cells exposed to 0.1% Triton X-100. Relative luminescence units (RLUs).

An assay based on lactate dehydrogenase release was performed to evaluate biomaterial cytotoxicity for RFP-HDFCs-Neo cells when exposed to chitosan-enriched biomaterials. The measurement was carried out using the luminescence method. The cells cultured on the biomaterials showed RLU (Relative Luminescence Unit) levels for the BCH/BCM positive control of biomaterials comparable to the positive control of adherent culture. The RLU levels were 14,906 ± 345.78, 15,720 ± 289.91, and 14,948 ± 458.2, respectively. For the negative control, where cells were cultured under standard conditions, the RLU level was 9913.5 ± 0.71. Luminescence levels were in accordance with the level for cell cultures on both BCH, where RLU = 9052.75 ± 301.06, and BCM, where RLU = 9673.25 ± 120.73. In the medium collected from the printed models, the positive control for both the cells printed with chitosan in biomaterial (BCH) and methacrylated chitosan (BCM) was comparable to the control of cells in adherent culture exposure to 0.1% Triton X-100; the RLU levels were 21,782 ± 687.3, 22,123 ± 24.0, and 21,737.7 ± 412.3, respectively. The highest level of RLU presented for BCH was 35,496.5 ± 7989.46. In the BCM model, the level of RLU was significantly less than for BCH, which was 14394 ± 1781.6. The BCH result was slightly more than for the negative control (cells cultured under standard conditions), RLU = 12,407.7 ± 737.37. On the basis of the statistical analysis carried out, no statistically significant differences were observed between the tested groups in the cell-seeded biomaterials (Figure 10A). For the 3D-printed models, statistically significant differences between BCH and BCM were observed at the level *p* < 0.0001 (Figure 10B).

#### 3.6.3. Cell Proliferation Assay

**Figure 11:**
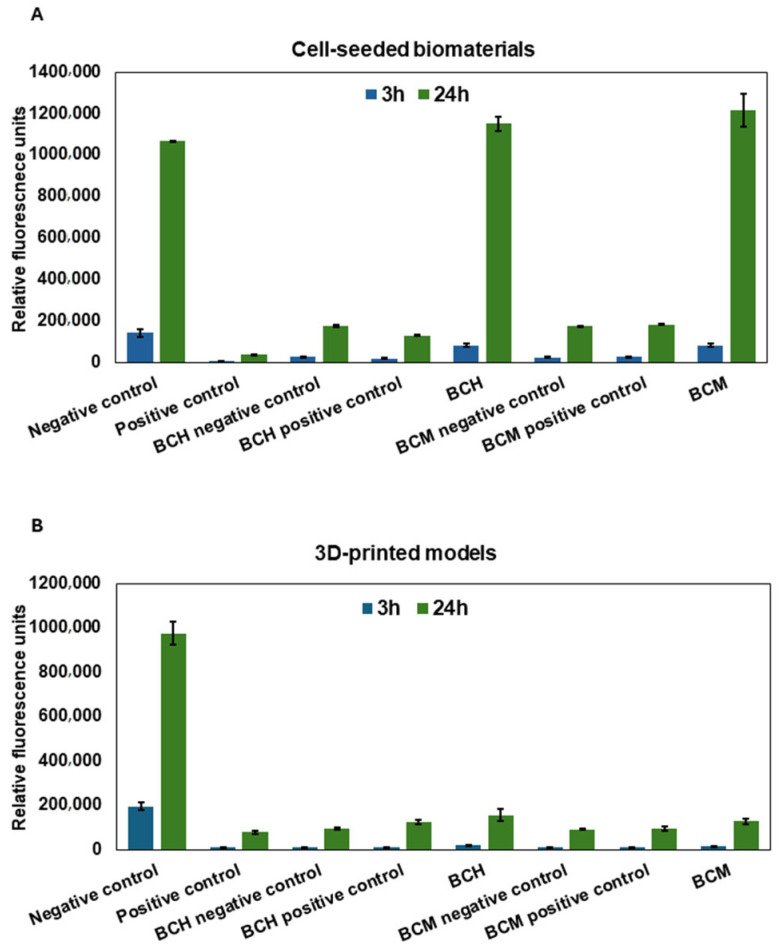
Cell proliferation as a result of exposure to biomaterials. (**A**) Cells cultured on the surface of biomaterials. (**B**) 3D-printed models. Negative control: cells cultured under standard conditions; positive control: cells cultured under standard conditions exposed to 0.1% Triton X-100; BCH: cells+ chitosan-biomaterial; BCM: cells+ methacrylated chitosan-biomaterial; BCH/BCM negative control: biomaterials without cells; and BCM/BCH positive control: biomaterials+ cells exposed to 0.1% Triton X-100. Relative fluorescence units (RFUs).

The commercially available Alamar Blue reagent was used to evaluate cell proliferation in the culture on biomaterials and after the printing process. The measurements were carried out at two time points: 3 h and 24 h after the seeding or the printing of the cell constructs. In both cell cultures on the surface of biomaterials (Figure 11A, Cell-seeded biomaterials) and 3D-printed models (Figure 11B, 3D-printed models), an increase in cell proliferation was observed within 24 h of the experiment. In particular, increased cell proliferation was observed within 24 h of exposure, specifically when cells were grown on the surface of biomaterials (Figure 11A). The cells cultured on the surface of BCH (after 3 h RFU = 80,786.25 ± 79,944.9; after 24 h RFU = 1,152,168 ± 34,419.13) and BCM (after 3 h RFU = 81,461.7 ± 9226.7; after 24 h RFU = 1,215,878 ± 81,097.37) show a comparable increase in proliferation compared to the negative control, which was cells cultured under standard conditions (after 3 h RFU = 142,986 ± 18,705.8; after 24 h RFU = 1,068,050 ± 193.8). The greatest level of proliferation was observed for cells cultured on biomaterial where the biomaterial was enriched with methacrylated chitosan (Figure 11A, BCM). For the 3D-printed models, there was no significant increase in proliferation over time compared to the negative control (after 3 h RFU = 194,864 ± 16,631.8; after 24 h RFU = 975,566 ± 52,365.9). For BCH, after the increase in proliferation, the RFU values were from 17,495 ± 1623.9 to 154,587.5 ± 27,060.3, and for BCM, the RFU values were from 12,970.7 ± 960.7 to RFU = 127,466.3 ± 13,370.6. For the cell-seeded biomaterials, statistically significant differences were observed between all groups at *p* < 0.0001 and for the BCH 24 h and BCM 24 h at *p* < 0.0451. For the BCH 3 h and BCM 3 h, no statistically significant differences were observed (Figure 11A). For the 3D-bioprinted models, all study groups demonstrated statistically significant differences at *p* < 0.0001 (Figure 11B).

#### 3.6.4. The Impact of Biomaterial Extracts on Cell Cytotoxicity

**Figure 12:**
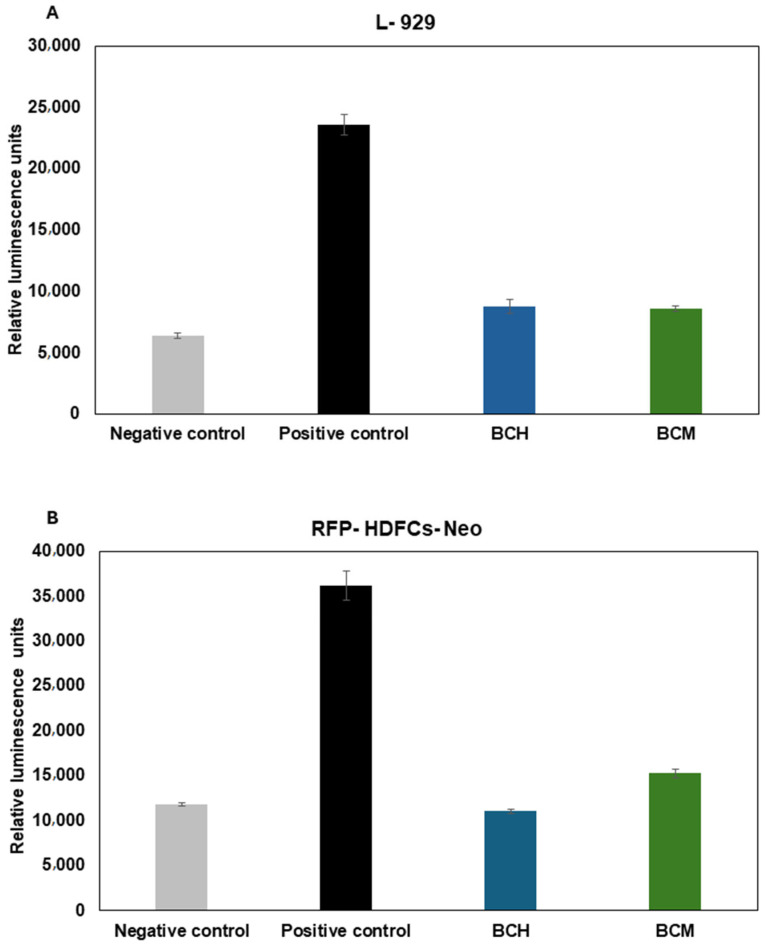
Cytotoxicity of biomaterial extract. LDH assay was performed with the use of L-929 (in accordance with standard PN-EN ISO 10993-5:2009) (**A**) and RFP-HDFCs-Neo (**B**) cells. Negative control: cells cultured under standard conditions (37 °C, 5% CO_2_); positive control: cells cultured under standard conditions (37 °C, 5% CO_2_) exposed to 0.1% Triton X-100; BCH: cells+ chitosan-biomaterial extracts; and BCM: cells+ methacrylated chitosan-biomaterial extracts.

A lactate dehydrogenase release-based assay was performed to assess the cytotoxicity of chitosan-enhanced biomaterial extracts to L-929 (Figure 12A) and RFP-HDFCs-Neo (Figure 12B) cells. The measurement was performed using the luminescence method. The highest lactate dehydrogenase release rate was observed for the positive control. It is estimated at 23571.8 ± 815.7 for L-929 cells and 36157.2 ± 1629.5 for RFP-HDFCs-Neo cells. The result of the analysis shows that in both cell lines, the cytotoxicity is at a similar level to that of the negative control and is estimated to be 6387.3 ± 216.9 for L-929 cells and 11831.2 ± 175.8 for RFP-HDFCs-Neo cells. For the cell-seeded biomaterials, no statistically significant differences were observed (Figure 12A). For the 3D-printed model, statistically significant differences between BCH and BCM were observed at *p* < 0.0001.

**Figure 13:**
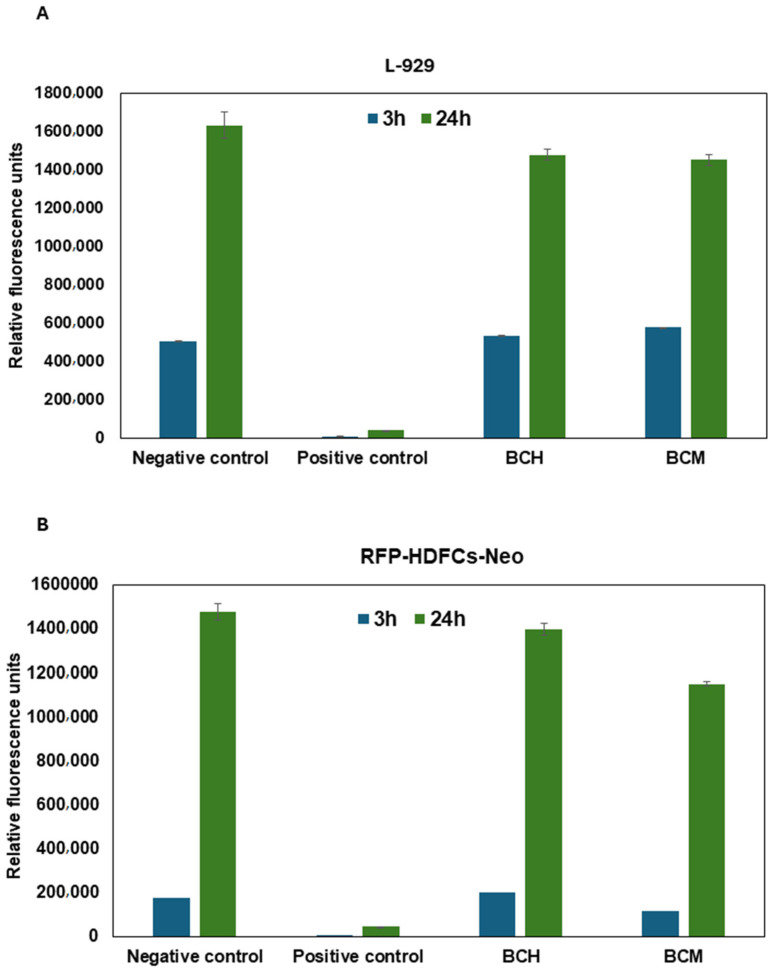
Cell proliferation after exposure to biomaterial extract. Alamar Blue assay was performed with the use of L-929 (in accordance with standard PN-EN ISO 10993-5:2009) (**A**) and RFP-HDFCs-Neo (**B**) cells. Negative control: cells cultured under standard conditions (37 °C, 5% CO_2_); positive control: cells cultured under standard conditions (37 °C, 5% CO_2_) exposed to 0.1% Triton X-100; BCH: cells+ chitosan-biomaterial extracts; and BCM: cells+ methacrylated chitosan-biomaterial extracts.

The cell proliferation was assessed at two time points, 3 and 24 h, after the application of the extracts to the cells. A significant increase in 24 h proliferation was observed for L-929 (Figure 13A) and RFP-HDFCs-Neo (Figure 13B) cells. A higher difference in the increase in proliferation per day was observed for the RFP-HDFCs-Neo cells. For the cell-seeded biomaterials, statistically significant differences were observed in all tested groups at *p* < 0.0001. For BCH 3 h and BCM 24 h, statistically significant differences were observed at *p* < 0.0419. No statistically significant differences were observed between BCH 24 h and BCM 24 h (Figure 13A). For the 3D-printed models, statistically significant differences were observed in all tested groups at *p* < 0.0001.

#### 3.6.5. Gene Expression Analysis

**Figure 14:**
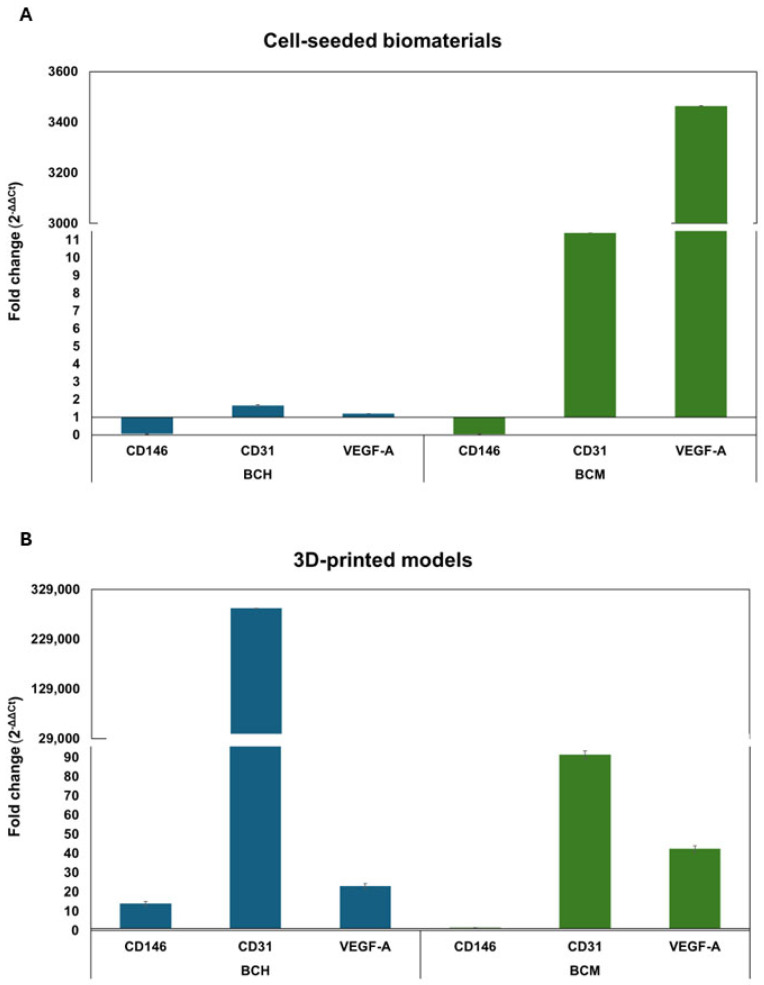
Expression of selected genes in RFP-HDFCs-Neo cells after exposure to biomaterials compared to cells cultured under standard conditions (defined as 1-fold change). (**A**) Gene expression of cells cultured on the surface of biomaterials; (**B**) gene expression of cells in 3D-printed models. BCH: cells+ chitosan-based biomaterial; BCM: cells+ methacrylated chitosan-based biomaterial.

This study investigated the expression of *CD146* (cell surface glycoprotein MUC18), CD31 (platelet endothelial adhesion molecule), and *VEGF-A* (vascular endothelial growth factor A) genes in cells after contact with biomaterials 24 h after exposure. All results were normalized to the *GAPDH* (Glyceraldehyde 3-phosphate dehydrogenase) reference gene. The control for the assay was cells cultured under standard conditions. The analysis was performed for both cell-seeded biomaterials (Figure 14A) and 3D-printed models (Figure 14B).

For cells cultured on chitosan-based biomaterial, the fold change in *CD31* (1.7 ± 0.05) and *VEGF-A* (1.2 ± 0.001) genes is similar to control cells defined as a 1-fold change. A decrease in fold change was observed for the *CD146* (0.1 ± 0.002) gene compared to the control cells. The analysis of the fold change in cells cultured on BCM shows different results. The *CD31* and *VEGF-A* genes are increased in the fold change in the mentioned genes, in particular regarding the *VEGF-A* gene (Figure 10A). Likewise for BCH, a decreased *CD146* fold change (0.05 ± 0.003) was observed for cells cultured on BCM compared to cells cultured under standard conditions. In the cells that were used in the bioprinting process, the change in the expression of *CD146*, *CD31*, and *VEGF-A* genes is significant (Figure 10B). There was an increased fold change in all the genes studied, in particular the *CD31* gene, in cells that were printed based on the BCH biomaterial. On the other hand, the cells used for BCM-based printing show a decrease in the fold change in the *CD31* gene compared to cells printed based on the BCH biomaterial. The fold change in the *CD146* gene for cells printed on the BCM biomaterial decreases compared to cells printed based on the BCH biomaterial (14.6 ± 1.15) and is 1.46 ± 0.16. This is near the fold change in the control cells (defined as a 1-fold change). For the VEGF-A gene, the fold change is greater than for cells printed based on the BCH biomaterial. Differences in the expression of individual genes could be related to the method of cell culture. Figure 14A represents cells seeded into the biomaterial, and Figure 14B shows cells mixed with the biomaterial and undergoing the printing process. In addition to the biomaterial itself, pressure and shear stress can also influence the changes in gene expression during printing.

## 4. Discussion

The ideal material for bioprinting should meet three main requirements: a relatively high viscosity, strong shear-thinning properties, and a fast cross-linking process after printing [55]. In addition to the requirements generated at the stage of material use, the ideal biomaterial in tissue engineering should be biocompatible, biodegradable, and guarantee a close fit to the mechanical properties of the replaced tissue and ensure cell adhesion. Recently, there has been an increase in interest in biomaterials that contain various additives intended to increase their functionality for their intended purpose. One such material worth considering may be chitosan. Chitosan is a polysaccharide of natural origin, often used in tissue engineering because, in addition to being biocompatible, non-toxic, and similar to the extracellular matrix, it decomposes into oligomers through lysozyme present in the human body [56]. Due to the possibility of using this polysaccharide as a biomaterial for 3D printing, chitosan solutions not only demonstrate stability in physiological conditions and appropriate viscosity values for bioprinting applications but also promote proper cell proliferation and differentiation [57]. In the context of chitosan cross-linking, it gels through both physical and chemical cross-linking mechanisms. However, a noticeable drawback in tissue engineering applications is the slow gelation rate associated with natural mechanisms. This limitation contrasts with the faster photocuring process. In its original form, chitosan is not photocurable. Nevertheless, as a result of recent progress, the use of UV light irradiation for chitosan gelation has been investigated, which is achieved by its prior chemical modification, among others, through methacrylation reactions. Modified chitosan enables an efficient and rapid cross-linking method, which solves the time challenges associated with traditional gelation mechanisms and increases its suitability for tissue engineering applications [58]. Methacrylated chitosan has the ability to quickly form mechanically stable networks in the photocuring process. There are several examples of research using methacrylated chitosan for biomedical purposes [59]. Nevertheless, despite already reported studies on photocross-linked chitosan scaffolds [60], 3D printing of stable chitosan-based structures is still challenging and under research [61].

This work compares the characteristics of useful materials containing chitosan and methacrylate chitosan. The features of biomaterials considered most important from the point of view of the usefulness of materials in 3D bioprinting technology were analyzed: rheological properties, printability, mechanical properties, degradation, and water absorption.

Biomaterials with significant utility in bioprinting must meet a number of requirements, including being printable and meeting the criteria for products used in tissue engineering [62]. The rheological properties of materials are very important in the context of their use in bioprinting technology to create models of tissues or organs. The biomaterial used in bioprinting should be viscous enough to ensure full reproduction of the shape of the printed model. However, it is necessary to ensure a balance between rheological properties and printability. Cells printed in a low-viscosity material have a more favorable environment for growth due to low stresses. On the other hand, the effect of the collapse of the printable material in three-dimensional space due to too low a viscosity of the biomaterial is well known [63]. These conclusions were also confirmed in our tests, where the BCM material had a much higher viscosity than the BCH material, which made it possible to produce a stable fiber printed on the platform and a lower diffusion rate, proving the high resolution of pattern printing.

The role of rheology in the development of new biomaterial compositions remains poorly understood and many studies do not consider rheology when developing and evaluating biomaterials. The vast majority of rheological characteristics of biomaterials focus only on the viscosity of the hydrogel [64]. The viscosity of the biomaterial can directly affect both the shape fidelity of the print and the printing pressure necessary to dispense the material, which, in turn, can affect cell viability; however, viscosity alone cannot capture the complex behavior of hydrogel-based biomaterials during the printing process [65,66].

It has been reported that the viscosity of the biomaterial is a determining factor in the shape fidelity of the print; however, high viscosity does not necessarily ensure high mechanical strength or printing accuracy (e.g., low-concentration hyaluronic acid) [67]. This results from two separate components of the dynamic modulus: the storage modulus (G′) and the loss modulus (G″) [68]. Until now, viscosity was considered a single parameter determining the proper composition of the biomaterial. Most studies have focused on reporting the G′ viscosity component for the specific type of hydrogel being developed, and the role of G′ is largely ignored [62,69]. The ratio between G′ and G′ is also important, which determines whether the material behaves like a solid or a liquid. The ratio of G″ to G′ is defined as the loss tangent (tanδ), and based on its value, the classification of viscoelastic materials is made [70]. In the case of the materials we analyzed, in the tested sample deformation range (1–100% amplitude), this ratio is between 0.1 and 1, which indicates properties corresponding to a viscoelastic solid with great utility in bioprinting technology in the context of maintaining the fidelity of the print shape [71]. High-viscosity hydrogels with an appropriate ratio between G′ and G′ are desirable to achieve good print resolution with sufficient mechanical strength to maintain structural integrity. However, higher-viscosity materials require higher extrusion forces. This may lead to the need to generate higher shear stresses affecting the cells and resulting in serious cell damage [69]. Additionally, research has shown that exposure to high levels of shear stress during the printing process can impact both immediate and long-term cell viability and proliferation.

The ideal biomaterial should be self-supporting and able to be squeezed through a thin nozzle. Typically, an increase in the biomaterial concentration results in an increase in the storage modulus and overall viscosity, while maintaining the loss modulus at an unchanged level. This suggests that the network formed by the material is highly elastic rather than viscous. Due to this feature, such hydrogels can be classified as stiff, solid biomaterial materials at room temperature, which seems to be ideal for the extrusion printing method. Similar relationships were obtained for our materials. From the point of view of using the material in bioprinting technology, it is important to analyze the material’s stability under the influence of stress. The LVR region is then determined depending on the deformation or stress modulus, which shows the range of stresses for which the material is stable. For evaluation, the curve of the G’ function is often preferred by users. In the LVR region, this function shows a constant value. In our experience, the BCM material is characterized by stability in a larger range of stresses, but at some point, a phase transition occurs, i.e., G″ > G′, which indicates a change in the nature of the sample. Additionally, interestingly, the modulus values for BCH are larger than for BCM, and the distance between the G′(γ) and G′(γ) relationships is larger than for BCM, which means that in this material the elastic properties significantly exceed the vicious ones. Strong shear thinning properties were demonstrated for BCM, which is a desirable feature for biomaterials used in 3D bioprinting. The G′ and G′ values of biomaterials can also predict the required extrusion pressure, which, in turn, may prove to be negatively related to cell viability. In the next course of research, the investigation should be extended to include a biological part to fully demonstrate the validity of the recommendation of the tested material for printing tissue models.

Generally, chitosan has quite poor mechanical properties. In order to improve its properties, the addition of other materials or chemical modifications, e.g., methacrylation, is used [72]. As shown in the literature, the biomaterial composition we developed, containing its methacrylate equivalent instead of chitosan, is characterized by high mechanical parameters, both strength and Young’s modulus. From the point of view of material use, an extremely important issue is to obtain a material with relatively good strength while ensuring an appropriate degradation rate [69]. Our materials have high mechanical strength and their degradation after 21 days is approximately 90%. The tested materials meet the requirements of biomaterials that can be used in systems exposed to significant shear stress, i.e., organ models with vascular systems and models of cartilage or bone. In addition, the materials used are printable at a very good resolution, making them applicable for printing models containing a lot of detail.

As well as the physicochemical parameters, the biological properties of the 3D-printed materials have also been tested [73,74]. In particular, it is important to test biomaterial cytotoxicity (Figure 8), cell proliferation (Figure 9), as well as cell functionality and biocompatibility as a result of biomaterial exposure (Figure 7, Figure 8, Figure 9 and Figure 10). This study used chitosan and methacrylated chitosan as an additive to biomaterial. The aim of the biomaterial modification was to increase the biocompatibility of the biomaterial in the target of RFP-HDFCs-Neo cells. To this purpose, a study was investigated, where cells were cultured directly on the BCH and BCM biomaterials or were mixed with the appropriate biomaterial variant (BCH or BCM) and subsequently 3D-printed. In this trial, after 24 h of culture on biomaterials and printed models, in the chitosan-containing biomaterials, particularly BCH, a large number of cells were observed that could be visualized under the microscope. It was found that bio-ink containing methacrylated chitosan (BCM) has an increasing effect on the proliferation of cells cultured on biomaterials, which was only noticeable after seven days of carrying out the experiment. For 3D-printed models, the effect of methacrylation results in the maintenance of a large number of cells throughout the experiment for both BCH and BCM (Figure 7). As in previously presented studies in this area, we also encountered a problem with the visualization of the cells. The main reason for this was the insufficient translucency of the biomaterial. Undoubtedly, the use of high-resolution electron microscopy may help to solve this problem in the future [68,69].

Based on the LDH release analysis study, it could be concluded that the chitosan-enriched biomaterial is biologically safe (Figure 10). The absence of toxicity to cells was observed both from contact with biomaterials (Figure 10A) and from cells included in 3D-printed models (Figure 10B). One can conclude that both chitosan and chitosan methacrylated as a biomaterial additive do not decrease the viability of RFP-HDFC-s-Neo fibroblast cells, except for BCH after printing (Figure 10A). The culture of cells on the surface of both BCH and BCM demonstrates that the level of LDH release is comparable to cells cultured under standard conditions (Figure 10A). For the 3D models, the BCM-printed constructs show lower levels of LDH release compared to the negative control (Figure 10B). Similar findings indicating the absence of toxicity associated with the use of chitosan as an additive to biomaterials were reported by Gheran, C.V. et al. [75]. The LDH assay was used in their study to evaluate the toxicity of the chitosan polymer matrix using mice macrophage cells. On the other hand, a study by Su, F. et al. [76] also highlights the potential use of chitosan addition to hydrogels. The team used, among other things, an assay involving the release of lactate dehydrogenase to evaluate the biocompatibility of chitosan-enriched hydrogels on the L-929 cells. In general, chitosan is considered a substance of high safety and low toxicity. In the context of evaluating the toxicity of methacrylate chitosan biomaterials, most researchers use other tests to assess cell viability than the LDH test [45,77,78]. For instance, Chen, C.C. et al. [78] showed that hydrogels with added methacrylate chitosan are also biosafe against the L-929 cells.

In our study, cell proliferation capacity was also assessed using the Alamar Blue assay (Figure 11). For cell culture on biomaterials for both BCH and BCM, cell proliferation increased over time compared to control cells (Figure 11A). For 3D-printed models, there was no such tendency (Figure 11B). Hussain, A. et al. [79] reported that chitosan addition in biomaterial increases the proliferation of cardiovascular cells, in particular fibroblasts and endothelial cells. Howling, G.I. et al. [80] demonstrate the relationship associated with increased cell proliferation and the addition of chitosan in cell culture in vitro. The researchers investigated the influence of chitosan derivatives on the proliferation of human skin fibroblasts and keratinocytes [57,75]. The analysis of their results indicates that chitosan modifications could impact cell mitogenesis. Moreover, the use of chitosan as an addition to increase cell proliferation could be used to rebuild skin after burning, for example. In addition, He, J. et al. [81] also presented that the addition of chitosan in hydrogels and its modification can increase stem cell proliferation. On the other hand, Patel, B. et al. [82] demonstrate that chitosan-based scaffolds support the mechanical enhancement of stem cell proliferation and differentiation. In addition, it is interesting to add that the hydrophilic structure of chitosan promotes the adhesion and proliferation of many types of cells. In particular, scaffolds allow chitosan to emerge as a suitable candidate for 3D organ bioprinting and could also be used for tissue repair. Celikkin, N. et al. [83] show that methacrylation influences cell proliferation. They investigated a study where researchers cultured human bone marrow mesenchymal stem cells encapsulated in GelMA. In this study, we observed not only the proliferation of osteogenic cells but also the induction of endothelial cell differentiation.

A laboratory study has suggested that chitosan may have some antibacterial and antiviral properties, which could be beneficial in the medical field. Nevertheless, it is necessary to recognize that the safety of chitosan could depend on the source from which it is obtained, as well as its purity. For biomedical applications, chitosan is being studied as a potential drug carrier, material for surgical sutures, and in the context of wound healing. As the study of chitosan progresses, it is necessary to consider the specific conditions of use and the type of product containing chitosan [76].

## 5. Conclusions

Chitosan is a very promising material that can be used to print tissue models. Generally, this material has quite poor rheological and mechanical properties, which is why it is often used as an additive to biomaterial or it is necessary to chemically modify it. The introduction of chemical modifications in the structure of the material in the form of methacrylic groups makes it very attractive in the context of using it in the bioprinting of tissue models because of its consistency and the possibility of fixing the printed structure change. This work presents the characteristics of biomaterials containing chitosan and its methacrylic equivalent in order to identify differences in their suitability in 3D bioprinting technology. The BCM material containing methacrylic chitosan has been shown to be three times more viscous than its non-methacrylated BCH counterpart. Additionally, the BCM material has good rheological properties, the storage modulus is stable in a larger range of stresses and has better printability parameters: resolution and fiber stability. The BCM material is characterized by higher mechanical parameters, both mechanical strength and Young’s modulus, than the BCH material. Both materials are ideal biomaterials for bioprinting, but BCM, due to its unique rheological properties and significant mechanical resistance, is a material recommended for creating tissue models that require resistance to high stresses, i.e., models with a vascular system or cartilage. Moreover, the addition of chitosan and especially its modification in methacrylation to the biomaterial promotes an increase in cell proliferation and reduces the toxicity of the biomaterial.

## 6. Patents

(P1) EP24163431 “Chitosan and methacrylated chitosan as biocomponents of bioink useful in tissue engineering and regenerative medicine” 14 March 2024.

## Data Availability

The original contributions presented in the study are included in the article, further inquiries can be directed to the corresponding authors.
